# Methylene Blue Instillation: A Cost-Effective Diagnostic Approach for Pleuroperitoneal Fistula in Resource-Limited Settings

**DOI:** 10.7759/cureus.69034

**Published:** 2024-09-09

**Authors:** José Manuel García Romero, Pedro Hugo Guerrero Morales, Ana Laura Alegria Arias, Daniela de Noriega Guzmán, Mariana Bulle Parra

**Affiliations:** 1 Transplant and Donation Department, Regional General Hospital 1 of the Mexican Social Security Institute, Querétaro, MEX; 2 General Practice, Health Clinic 56 Amealco of the Mexican Social Security Institute, Querétaro, MEX; 3 General Practice, Health Clinic 11 Bicentenario of the Mexican Social Security Institute, Querétaro, MEX; 4 General Practice, Universidad Anáhuac Querétaro, Querétaro, MEX

**Keywords:** fistula, hydrothorax, methylene blue infusion, peritoneal dialysis, pleuroperitoneal leak

## Abstract

A pleuroperitoneal fistula, an uncommon complication of peritoneal dialysis, involves a connection between the peritoneal cavity and the pleural space. This case highlights a cost-effective diagnostic option for detecting these fistulas in primary care hospitals and emphasizes the importance of considering this condition in patients with a history of peritoneal dialysis and persistent pleural effusion. We present a case of a 55-year-old female patient undergoing peritoneal dialysis for end-stage renal disease who developed a pleuroperitoneal fistula, leading to persistent pleural effusion. This condition was successfully diagnosed using methylene blue instillation into the dialysis bags, demonstrating a potentially viable diagnostic technique that is effective, safe, and cost-efficient for primary and secondary care hospitals. The presented case underscores that pleural effusion, as a complication with high mortality, should be considered in any patient with end-stage renal disease presenting with dyspnea, desaturation, and pleuritic pain secondary to peritoneal dialysis sessions. Furthermore, the use of methylene blue is proposed as a cost-effective and accessible diagnostic alternative. This approach not only facilitates accurate diagnosis but also simplifies the diagnostic process without relying on advanced imaging technologies that might not be available.

## Introduction

Pleuroperitoneal fistula (PPF) is a rare but significant complication that can occur in patients undergoing peritoneal dialysis, a treatment used for end-stage renal disease (ESRD). First described in 1967, PPF occurs when there is an abnormal connection between the peritoneal cavity (the space within the abdomen) and the pleural space (the cavity surrounding the lungs). This can lead to fluid migrating from the abdomen into the chest, causing pleural effusion, which can be life-threatening due to the pressure it places on the lungs [1].

Peritoneal dialysis (PD) is a treatment for kidney failure that involves introducing a special fluid into the abdomen to help remove waste products and excess fluids from the body. This process relies on the peritoneum, a membrane lining the abdominal cavity, to facilitate the exchange of substances between the blood and the dialysis fluid [2]. Although PD is effective and convenient, it can sometimes lead to complications. One such complication is a pleuroperitoneal fistula, an abnormal connection between the abdominal and chest cavities that can cause significant issues with breathing and fluid balance. Understanding the underlying mechanisms, clinical implications, and management strategies for this complication is crucial for optimizing patient outcomes and improving quality of life [3].

The development of a pleuroperitoneal fistula is often linked to increased pressure within the abdomen, which can damage the diaphragm (the muscle separating the chest and abdomen) and create a direct link between the two cavities. This can occur due to high volumes of dialysis fluid, repeated changes in abdominal pressure, or weaknesses in the diaphragm. When a PPF forms, dialysis fluid can enter the chest cavity, leading to fluid accumulation in the lungs (hydrothorax). This can cause breathing difficulties, reduced lung function, and other related problems [4].

Diagnosing pleuroperitoneal fistulas can be challenging due to their rarity and the potential overlap with other conditions that cause hydrothorax. Patients undergoing PD who suddenly experience shortness of breath, a persistent cough, or worsening respiratory function should be evaluated further. Diagnostic approaches typically include imaging studies like chest X-rays or CT scans, which can reveal fluid accumulation in the pleural space and potentially identify the presence of a fistulous connection [5].

Management of pleuroperitoneal fistulas involves a combination of conservative and interventional strategies. Initial management often focuses on conservative measures, including modifications to the dialysis regimen to reduce intra-abdominal pressure and optimizing fluid balance. This may include decreasing dialysate volume or frequency of exchanges. In cases where conservative measures are insufficient, more invasive approaches such as surgical repair of the fistula or placement of a pleural drain may be necessary. The goal is to alleviate symptoms, prevent complications, and restore normal respiratory function [6].

In this case report, we discuss a patient diagnosed with pleural effusion who underwent a diagnostic procedure involving methylene blue instillation in the dialysis fluid to identify the presence of a pleuroperitoneal fistula.

## Case presentation

A 55-year-old female patient with end-stage renal disease began peritoneal dialysis in 2022 and experienced complications, including catheter migration and peritonitis, which necessitated repositioning of the Tenckhoff catheter twice that year. Initially, she was on continuous ambulatory peritoneal dialysis using a 2.5% solution with four exchanges per day of 1250 ml.

**Figure 1:**
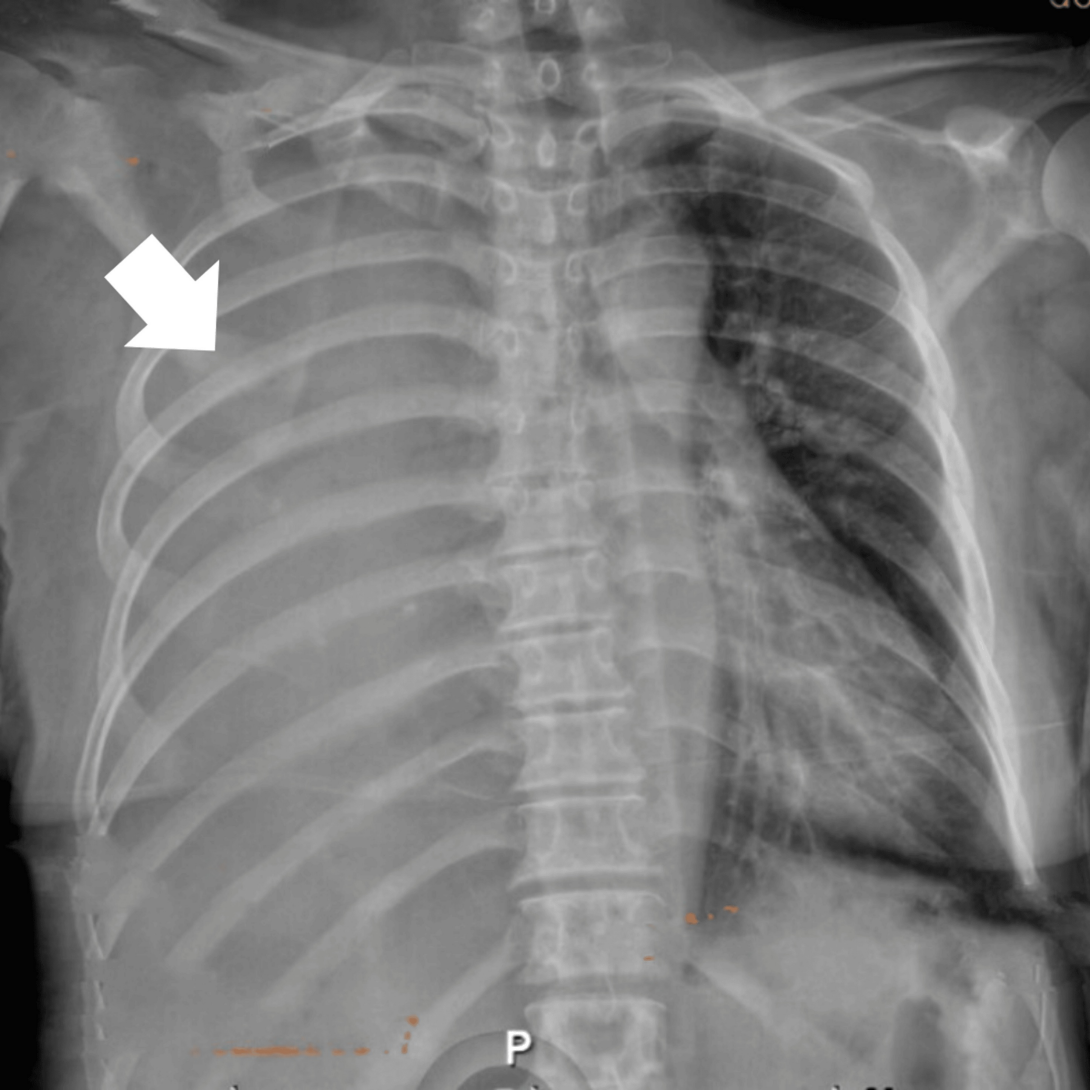
Chest X-ray upon admission showing massive hydrothorax involving 100% of the right hemithorax Anteroposterior chest X-ray showing increased radiopacity in the right hemithorax (white arrow) due to the occupation of the pleural space by fluid-dense tissue.

In January 2023, the patient presented with worsened dyspnea and oxygen desaturation to 88%, requiring home oxygen therapy at 2 L/min. Her medical history included a hospitalization in December 2022 for community-acquired pneumonia complicated by right parapneumonic effusion. Upon admission to the emergency department, she was found to be in respiratory distress with a respiratory rate of 22 breaths per minute and oxygen saturation of 96% on a nasal cannula at 3 L/min. Physical examination revealed decreased respiratory movement and generalized dullness on percussion of the right hemithorax. A chest X-ray revealed a complete right hemithorax pleural effusion, prompting a diagnostic thoracentesis that drained 500 cc of fluid (Figure 1). 

This fluid was analyzed using Light's criteria. The laboratory report indicated that the fluid did not meet the criteria for an exudate, showing no malignant cells or bacteria, a slight increase in glucose concentration, and low levels of lactate dehydrogenase (LDH) and proteins (Table 1).

**Table 1:** Laboratory values from the cytochemical analysis of drained pleural fluid along with blood values. Results of the cytochemical analysis of the pleural fluid showing an increase in urea levels in the pleural fluid, as well as LDH (lactate dehydrogenase), glucose, and protein levels that do not meet Light's criteria to define the pleural fluid as an exudate.

	Value	Reference Range
Pleural fluid glucose (mg/dL)	166	(60 - 100)
Pleural fluid LDH (U/L)	70	(< 200 - 300)
Pleural fluid urea (mg/dL)	79	(10 - 30)
Pleural fluid proteins mg/dL)	632	(2500 -3000)
Blood glucose (mg/dL)	90	(74 - 106)
Blood LDH (U/L)	228	(125 - 220)
Blood urea (mg/dL)	68.48	(20 - 43)

**Figure 2:**
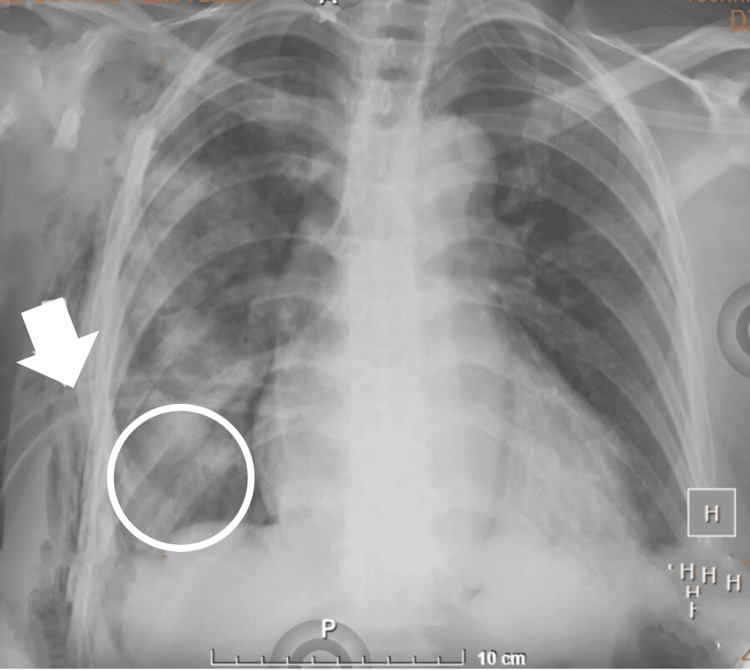
Chest X-ray after placement of an endopleural seal for drainage of massive hydrothorax Anteroposterior chest X-ray showing persistent occupation of the pleural space by fluid-dense tissue (white circle). The course of the endopleural tube is also indicated (white arrow).

However, the high urea level of 79 mg/dL suggested the possibility of communication between the peritoneal and pleural cavities. This elevated urea level could be explained by peritoneal dialysis fluid entering the pleural space. Given the persistent pleural effusion, an endopleural seal was placed, leading to progressive improvement with a total of 3500 ml drained over the subsequent days (Figure 2).

**Figure 3:**
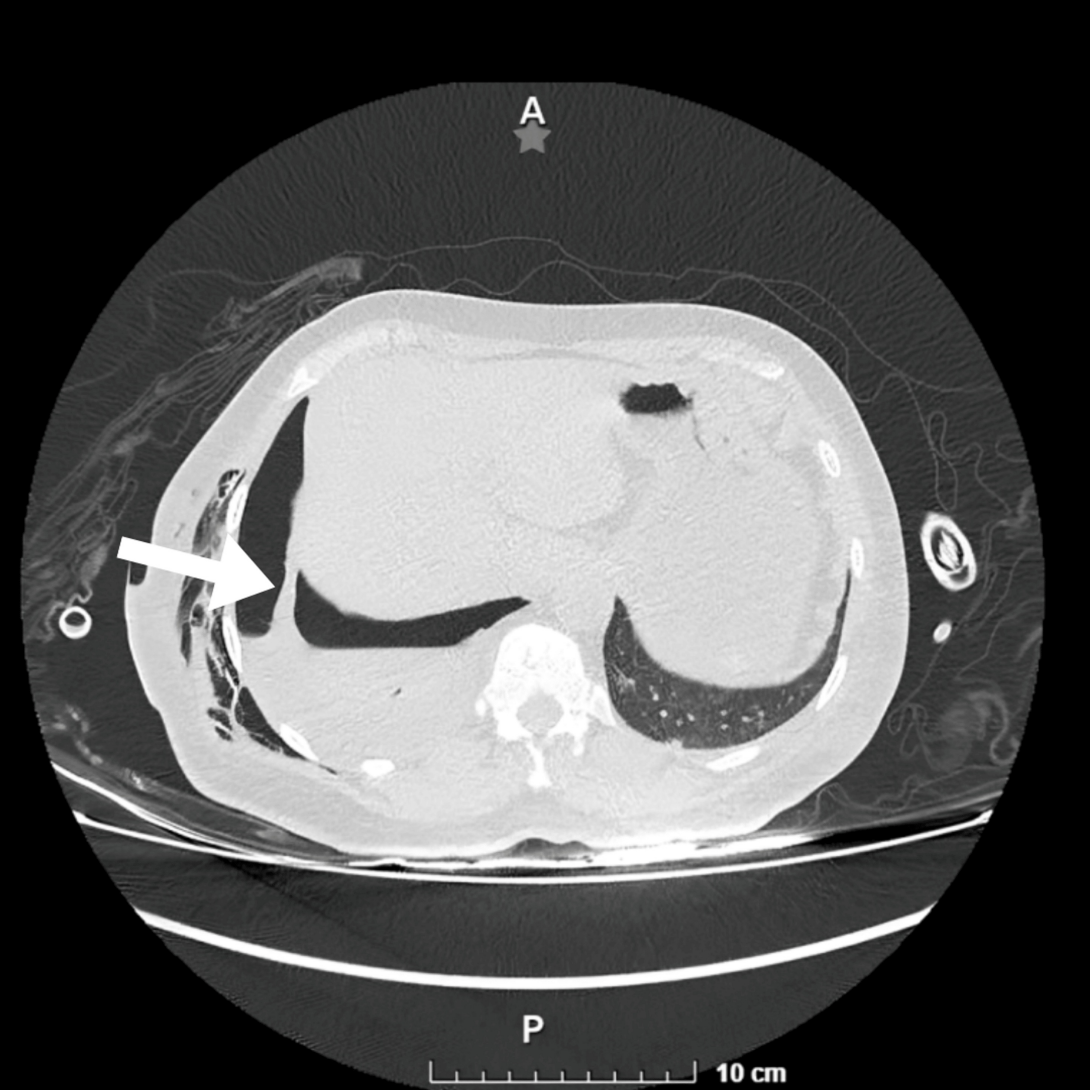
Computed tomography (CT) of the thoracoabdominal area in pulmonary window CT scan showing communication between the pleural and peritoneal cavities in the right hemithorax (white arrow)

The properties of the fluid, combined with the patient’s history of chronic peritoneal dialysis, variations in pleuro-peritoneal pressure gradients, and a prior inflammatory episode during pneumonia, raised the suspicion of a pleuroperitoneal fistula. To confirm this, a methylene blue instillation test was performed, infusing 2 mL of methylene blue, equivalent to 20 mg, into the peritoneal dialysis fluid bag. The presence of dye was detected in the endopleural fluid two hours later, confirming the communication between the cavities. A chest and abdominal CT scan further verified this diagnosis by showing a connection between the pleural effusion and the hepatic capsule (Figure 3).

**Figure 4:**
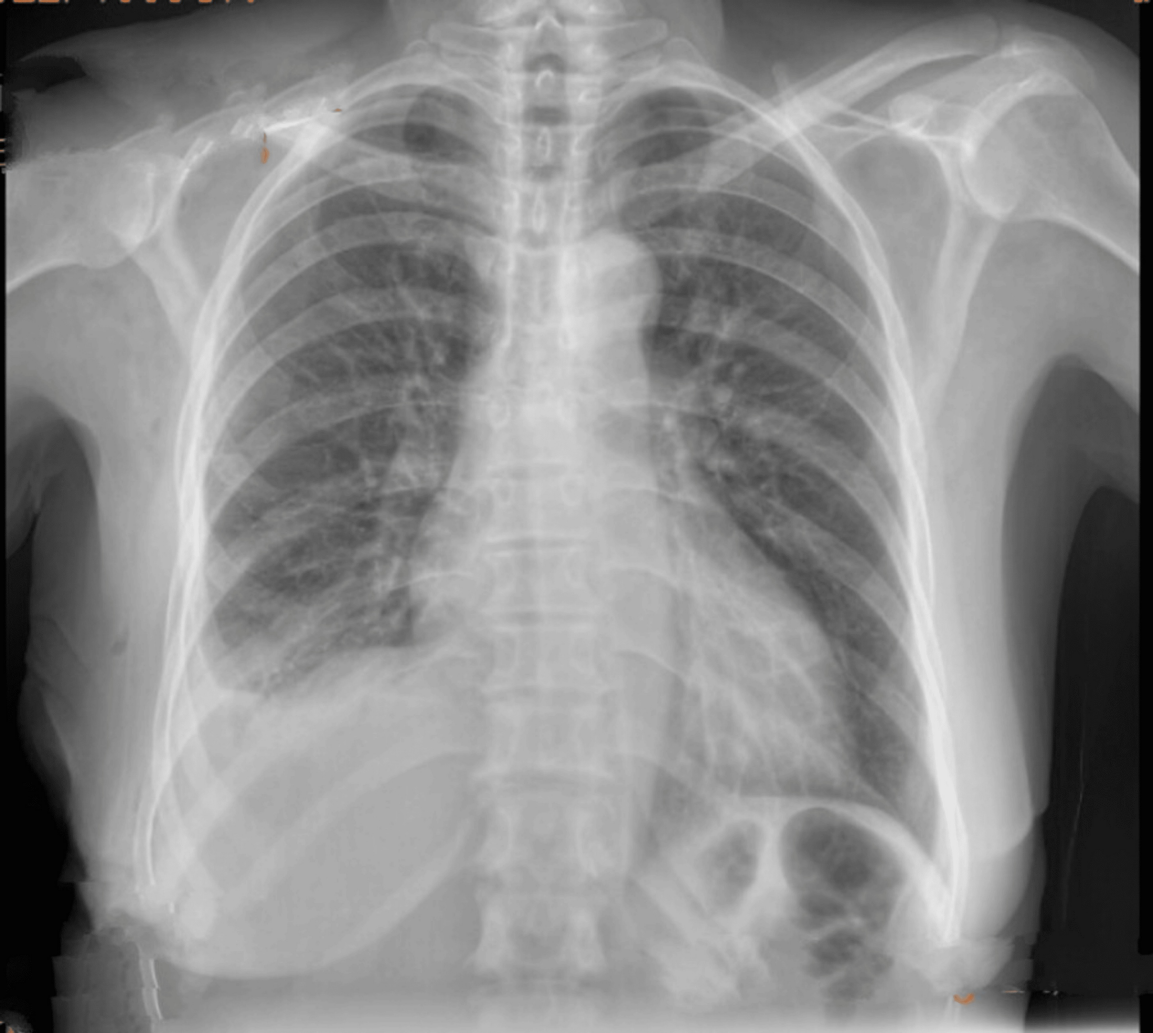
Chest X-ray at discharge showing complete resolution of the hydrothorax.

Given the confirmed diagnosis of a pleuroperitoneal fistula, the Tenckhoff catheter was removed and replaced with a Mahurkar catheter for permanent hemodialysis. The pleural effusion resolved completely by discharge (Figure 4).

To date, in the quarterly reviews at the nephrology service, the patient has shown adequate clinical progress, with no hospitalization events or symptoms suggesting recurrence of the pleuroperitoneal fistula or complications associated with the use of methylene blue, such as chemical peritonitis. The patient is currently continuing hemodialysis with good adherence to her sessions.

## Discussion

Peritoneal dialysis is commonly used as a renal replacement therapy. Among the complications that can arise from this procedure are infectious peritonitis, peritoneal sclerosis, catheter leaks or obstruction, abdominal wall hernias, and intestinal perforation, particularly during catheter placement [5].

Pleuroperitoneal fistula (PPF), an extremely rare complication of continuous ambulatory peritoneal dialysis, has an estimated incidence between 0.64% and 6%. This condition predisposes fluid migration from the peritoneal cavity to the pleural space through fistulas, which can lead to the development of acute pleural effusion [6].

Without an established etiological process, proposed theories to explain the development of a pleuro-peritoneal communication focus on congenital diaphragmatic structural defects, muscle hypotonia secondary to collagen fiber loss in the diaphragm due to altered pleuro-peritoneal pressure gradient with a constant increase in intra-abdominal pressure during peritoneal dialysis, or lymphatic drainage alterations due to long-term overload [7].

Other causes include abdominal or thoracic trauma, prior surgeries involving both the abdominal and thoracic cavities and abdominal or thoracic infections that can erode through the diaphragm [8].In the present case, one hypothesis for the development of the pleuroperitoneal fistula centers on the history of bacterial pneumonia. It is hypothesized that the pneumonia may have contributed to the weakening of the diaphragmatic membrane since both the pneumonia and the wall defect occurred in the right hemidiaphragm. The inflammatory process and possibly the presence of infection in the diaphragm may have caused erosion or a defect in this membrane, creating an abnormal communication between the two body spaces [9].

Clinical data in a patient developing pleural effusion are often asymptomatic or present late. In our patient, the clinical presentation was characterized by dyspnea, pleuritic pain, tachycardia, and desaturation. Diagnosing pleural effusion due to PPF should integrate both biochemical and imaging tests [10]. Diagnostic thoracentesis typically shows glucose concentrations in pleural fluid higher than serum levels [11].

Methylene blue instillation has been employed as a diagnostic tool for various types of fistulous communications, including gastrointestinal, broncho-pleural, and vesicovaginal fistulas. This technique is particularly noted for its utility in identifying rectovaginal and enterocutaneous fistulas due to its ability to visibly trace the flow of the dye through abnormal connections [12]. In the case presented, methylene blue was used in peritoneal dialysis bags to reveal the communication between the peritoneal and pleural cavities by detecting the dye in the fluid drained from the endopleural seal, offering a novel approach to diagnosing pleuroperitoneal fistulas. The effectiveness of methylene blue in diagnosing gastrointestinal fistulas is well-documented, with its use in identifying colovesical fistulas by instilling the dye into the colon and observing its appearance in the urinary tract [13]. For broncho-pleural fistulas, methylene blue has been used to trace connections between the bronchial tree and pleural space, with studies highlighting its utility in difficult diagnostic cases [14, 15].

A small cohort study by Sakata et al., demonstrated that controlled doses of methylene blue can significantly minimize associated complications. In their study, a single milliliter of methylene blue, containing 10 mg, was diluted 250-fold with normal saline for intrapleural administration. This approach resulted in minimal exposure to the dye, as only the necessary amount was used to visualize the dye emitting into the bronchi. Notably, the procedure involved instilling approximately 80 mL of the diluted solution per case, thereby exposing patients to an exceedingly small quantity of methylene blue. Furthermore, all chest tubes were promptly reconnected to suction post-procedure to evacuate the pleural space and limit any potential systemic effects. The study did not report adverse events commonly associated with methylene blue, such as pleuritis, pneumonitis, serotonin syndrome, or hemolysis, especially in patients with G6PD deficiency. This careful management of dosage and prompt post-procedure measures helped mitigate risks and suggest that methylene blue when used in controlled amounts can be a safe and effective diagnostic tool for localized applications [14]. In our study, we used 2 mL of a 1% methylene blue solution, which remains a low dose compared to the amounts used for treating conditions such as methemoglobinemia and vasoplegia, where accumulated doses can reach up to 300 mg [14]. Therefore, the risk of complications is inversely proportional to the concentration employed for diagnosing the fistulous tract.

Peritoneal tomography is an effective option for visualizing the peritoneal cavity and is more accessible than magnetic resonance imaging or single-photon emission computed tomography (SPECT)/CT [16]. Another frequently described option is peritoneal scintigraphy with technetium-99m macroaggregated albumin (99mTc-MAA) as a contrast agent, although some reports indicate that its sensitivity rarely exceeds 50% [17]. These methods are highly sensitive and effective for identifying pleuroperitoneal fistulas and the resulting pleural effusion, but their implementation is limited in medical contexts with restricted resources, as is common in primary care hospitals.

Spontaneous resolution cases are rare, and therapeutic intervention has not been established through consensus due to the infrequency of this complication [18]. Initial measures include suspending peritoneal dialysis to avoid worsening the pleural effusion, and in certain cases, performing therapeutic thoracentesis with pleurodesis [19,20]. Regarding invasive interventions, surgical pleurectomy, mechanical abrasion, and diaphragmatic patching have shown high efficacy [21]. However, the management of our patient involved changing the renal replacement therapy modality to hemodialysis.

The identification of a pleuroperitoneal fistula was supported by the elevated urea level in the pleural fluid, indicating a communication between the peritoneal and pleural cavities. This biochemical evidence was corroborated by the methylene blue instillation test and CT imaging, which confirmed the diagnosis by visualizing the connection between the cavities. The accurate diagnosis guided the management decisions, leading to the removal of the Tenckhoff catheter and the transition to hemodialysis, which resulted in complete resolution of the pleural effusion and stabilization of the patient's condition. By linking these diagnostic steps directly to clinical improvement and the strategic shift in renal replacement therapy, the text underscores the practical significance of the diagnostic process and its role in effective patient management. This approach not only validates the diagnostic methods but also highlights their critical role in informing treatment decisions and improving patient outcomes.

## Conclusions

The presented case highlights the proposal to use methylene blue as a cost-effective and accessible diagnostic alternative. This technique, being relatively simple and requiring minimal resources, allows for the direct visualization of communication between the pleural and peritoneal cavities by coloring the pleural fluid. This not only facilitates accurate diagnosis but also simplifies the diagnostic process without relying on advanced imaging technologies that might not be available.

While methylene blue instillation presents a promising and cost-effective diagnostic tool for identifying various types of fistulas, including gastrointestinal, broncho-pleural, and vesicogenital, it is essential to acknowledge the limitations of this study. This report is a single-case study, which underscores the need for further research to establish methylene blue instillation as a standard diagnostic tool for pleuroperitoneal fistulas. Future investigations should focus on evaluating long-term outcomes, peritoneal absorption rates, complication rates, and the overall efficacy of methylene blue in diagnosing fistulas across different settings. Additionally, more comprehensive studies could assess the potential advantages of this technique in low-resource hospital environments, where cost-effectiveness and logistical feasibility are critical. The promise of methylene blue lies in its potential to provide a valuable diagnostic option, especially where advanced diagnostic technologies are not readily available.
